# Spontaneous Self‐Assembly of Cesium Lead Halide Perovskite Nanoplatelets into Cuboid Crystals with High Intensity Blue Emission

**DOI:** 10.1002/advs.201900462

**Published:** 2019-05-08

**Authors:** Chenghao Bi, Shixun Wang, Stephen V. Kershaw, Kaibo Zheng, Tönu Pullerits, Sergey Gaponenko, Jianjun Tian, Andrey L. Rogach

**Affiliations:** ^1^ Institute for Advanced Materials and Technology University of Science and Technology Beijing 100083 China; ^2^ Department of Materials Science and Engineering and Centre for Functional Photonics (CFP) City University of Hong Kong Kowloon Hong Kong S.A.R.; ^3^ Department of Chemical Physics and NanoLund Lund University P. O. Box 124 22100 Lund Sweden; ^4^ B. I. Stepanov Institute of Physics National Academy of Sciences of Belarus 68 Nezaležnasci Ave., 220072 Minsk Belarus

**Keywords:** blue emission, cesium lead halide perovskite, nanoplatelets, quantum wells, self‐assembly

## Abstract

Colloidal all‐inorganic perovskite nanocrystals have gained significant attention as a promising material for both fundamental and applied research due to their excellent emission properties. However, reported photoluminescence quantum yields (PL QYs) of blue‐emitting perovskite nanocrystals are rather low, mostly due to the fact that the high energy excitons for such wide bandgap materials are easily captured by interband traps, and then decay nonradiatively. In this work, it is demonstrated how to tackle this issue, performing self‐assembly of 2D perovskite nanoplatelets into larger size (≈50 nm × 50 nm × 20 nm) cuboid crystals. In these structures, 2D nanoplatelets being isolated from each other within the cuboidal scaffold by organic ligands constitute multiple quantum wells, where exciton localization on potential disorder sites helps them to bypass nonradiative channels present in other platelets. As a result, the cuboid crystals show an extremely high PL QY of 91% of the emission band centered at 480 nm. Moreover, using the same synthetic method, mixed‐anion CsPb(Br/Cl)_3_ cuboid crystals with blue emission peaks ranging from 452 to 470 nm, and still high PL QYs in the range of 72–83% are produced.

## 1 Introduction

Motivated by the increasing demands for lighting and display applications, all‐inorganic perovskite nanocrystals (NCs) of the general formula CsPbX_3_ (X = Cl, Br, and I) have been explored extensively in terms of their cost‐effective chemical synthesis, narrow emission band widths offering high color purity, size‐ and composition‐tunable emission spectra, and often high photoluminescence quantum yield (PL QY).^1^ The superior light‐emission of CsPbBr_3_ NCs has been recently explained by the reverse order of the exciton fine‐structure levels, where owing to the Rashba effect the lower three levels are expected to be bright excitons.^2^ Compared with traditional metal chalcogenide NCs, metal halide perovskite NCs often display high PL QYs even without any additional passivating shell coating, which is commonly explained by their “defect‐tolerant” nature.^3^ Bromide‐ and iodide‐based CsPbX_3_ perovskite NCs with PL QY exceeding 90% were reported, such as green‐emitting CsPbBr_3_ NCs with PL QY of 95%,^4^ and red‐emitting CsPbI_3_ NCs with nearly 100% PL QY.^5^ However, it proved to be challenging so far to synthesize blue‐emitting CsPbX_3_ with such high PL QYs. One reason for that is that for the wide bandgap (blue emitting) nanomaterials, high energy excitons are easily captured by interband traps, resulting in a decrease of the PL QY. Blue‐emitting perovskite NCs have been usually synthesized by matching the proportion of Br^−^ and Cl^−^ ions in the mixed‐halide CsPb(Br/Cl)_3_ NCs.[qv: 1h,4,6] For these mixed‐halide NCs, however, PL QY did not exceed 40%, also due to the lattice mismatch.^4^ Therefore, the external quantum efficiency (EQE) of blue light emitting diodes (LEDs) based on such mixed‐halide NCs was only around 1%,[qv: 1a,b,7] far lower than the EQEs of LEDs utilizing green‐emitting CsPbBr_3_ NCs (>10%),^8^ or red‐emitting CsPbI_3_ (>11%).[qv: 1j] In order to realize higher PL QY of the blue‐emitting perovskite NCs, the introduction of divalent or trivalent cations into the lattice of CsPbBr_3_ nanoparticles has been attempted. van der Stam et al. used postsynthetic cation exchange reactions in CsPbBr_3_ NCs, partially replacing Pb^2+^ cations by divalent cations (M = Sn^2+^, Cd^2+^, and Zn^2+^), and reported samples with blue emission (at 479 nm) with PL QY of 62%.^9^ We recently reported a high blue PL QY (over 80%) from core/shell structured CsPbBr_3_ NCs, consisted of a 2 nm size core (blue emitting due to the quantum confinement effect) and an amorphous CsPbBr*_x_* shell (passivating layer).[qv: 1k] While this was one of the highest PL QYs for the blue‐emitting inorganic perovskite NCs reported in literature, the amorphous shell had rather poor thermal stability.[qv: 1k] So far, the emission performance of blue‐emitting perovskite NCs still lags well behind the requirement for applications like solid state lighting and displays.

Blue emission can also be realized by controlling the size and the shape of CsPbBr_3_ NCs, namely through the formation of ultrathin nanoplatelets (NPLs).^10^ Liang et al. reported 2D CsPbBr_3_ NPLs with a thickness of 2.5 nm, which had blue emission at 449 nm with a PL QY of 54%. However, the abundance of surface traps of individual 2D NPLs still results in higher nonradiative recombination rates, negatively impacting their PL QY. In this work, we show how to tackle this issue through the spontaneous self‐assembly of CsPbBr_3_ NPLs into larger (50 nm × 50 nm × 20 nm) cuboidal‐shaped perovskite NCs consisting of multiple quantum wells, which keep the original blue emission of the 2D NPLs, but which experience much lower surface trap density, leading to a record high PL QY reaching 91%. The same synthetic method has been used to produce mixed‐anion CsPb(Br/Cl)_3_ cuboid crystals with emission peaks centered between 452 and 470 nm, and still high PL QYs in the range of 72–83%.

## 2 Results and Discussion

**Figure 1:**
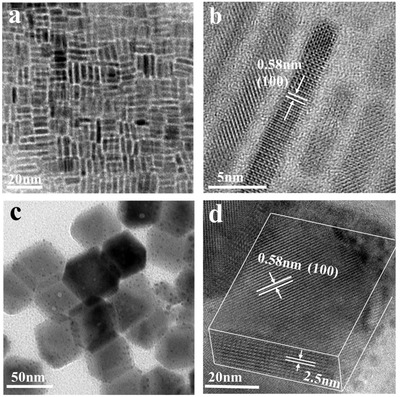
TEM and HRTEM images of a,b) CsPbBr_3_ NPLs and c,d) CsPbBr_3_ cuboid NCs.

To trigger spontaneous self‐assembly of 2D perovskite NPLs into larger particles during their formation and growth, the conventional organic ligand oleylamine (OAm) was replaced with a shorter carbon‐chain ligand octylamine (OTA) in their hot injection synthesis. In addition, the molar ratio of OTA to oleic acid (OA), and the reaction time were adjusted to achieve the best results. **Figure** **1** shows the appearance of the reaction products formed at two different stages of the ongoing reaction quenched using an ice bath after the injection of Cs‐oleate (see the Supporting Information for the experimental details). As shown in Figure 1a,b, a considerable amount of face‐to‐face stacking 2D NPLs were formed at 10s after injecting the Cs‐oleate. The thickness of the CsPbBr_3_ NPLs is about 2 nm, which corresponds to 2–3 unit cells, in accordance with the optical data as will be discussed below. Well resolved lattice fringes throughout the whole NPLs, with an interplanar distance of 0.58 nm, corresponding to the (101) crystal lattice planes of CsPbBr_3_, indicating that the growth direction occurs along {101} direction. As the reaction time increased to 20s, the 2D NPLs underwent self‐assembly into multiple‐stack rectangular solids with some amount of cuboid‐shaped crystals, as shown in Figure S1 of the Supporting Information. After 30s, regular‐sized, cuboid‐shaped CsPbBr_3_ NCs with sizes of around 50 nm × 50 nm × 20 nm were obtained as shown in Figure 1c,d and Figure S2 (Supporting Information), which is consistent with the size histograms (Figure S3, Supporting Information) and X‐ray diffraction (XRD) refinement results (Table S1, Supporting Information). Their lattice fringes with an interplanar distance of 0.58 nm correspond to the (101) lattice plane of the orthorhombic CsPbBr_3_ structure.^11^ The periodic stacked structure with a repeating distance of 2.5 nm can be observed on the underside of the cuboid, indicating that the cuboid NCs consist of the multiple adjacent NPLs. Due to the comparatively short length of the OTA ligands between adjacent NPLs, the distance between them remains rather short, and hence, the cuboid NCs appear in transmission electron microscopy (TEM) as large 3D single crystals shown in Figure 1d.

**Figure 2:**
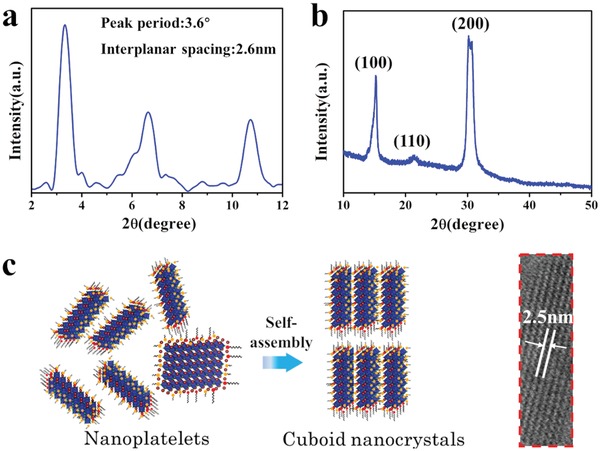
a) Small‐angle XRD pattern of CsPbBr_3_ cuboid NCs. b) Wide‐angle XRD pattern of CsPbBr_3_ cuboid NCs. c) Schematic diagram of the formation of CsPbBr_3_ cuboid NCs through the spontaneous self‐assembly of NPLs. An inset in (c) provides a closer view of a magnified area of a single cuboid as shown in Figure 1d.

The XRD analysis was carried out to further verify the multiple quantum wells structure of the CsPbBr_3_ cuboid NCs (**Figure** **2**). The structure of the cuboid NCs consisting of multiple NPLs is directly confirmed by small‐angle X‐ray diffraction measurement, as shown in Figure 2a. At small angles, a series of diffraction peaks originating from the stacked NPLs of the cuboid NCs was observed. The periodic peaks indicate a stacking distance of 2.6 nm for adjacent NPLs within the cuboid crystals, which is in line with the high‐resolution TEM (HRTEM) data as shown in Figure 1d. An effective distance of organic ligand layers with ≈0.3 nm can be calculated by subtracting inorganic layers. These results provide a direct evidence that the cuboid NCs consist of the 2D perovskite NPLs (with Cs:Pb:Br ratio of 1:1.02:3.03, according to X‐ray photoelectron spectroscopy (XPS) analysis in Table S3, Supporting Information) forming multiple quantum wells surrounded by organic ligands providing an appropriate separation between them. The wide‐angle XRD pattern of the CsPbBr_3_ cuboid NCs (Figure 2b) shows diffraction peaks at 2θ = 15.1°, 22.1°, and 30.2° corresponding to (100), (110), and (200) planes of orthorhombic CsPbBr_3_ (JCPDF #01‐072‐7929), respectively, with space group *Pbnm* (62). There are two apparent diffraction peaks at 2θ = 15.1° and 30.2° in the XRD spectra of the CsPbBr_3_ NPLs (Figure S4, Supporting Information). Compared to NPLs, there is a more obvious diffraction peak at 22.1° in cuboid NCs XRD pattern, which is corresponding to (110) plane. It can be attributed to oriented growth of perovskite crystals induced by oleic acid, which has a strong binding ability with (001) crystal facets of CsPbBr_3_ perovskites, as well as the result of the self‐assembly process, leading to their growth along (110) crystal plane.^12^ During self‐assembly process (insulation stage), the most of oleic acid ligands were removed and the short chain OTA ligands remained, due to that the binding energy with perovskite of OTA is much stronger than that of oleic acid. This can be verified by Fourier transform infrared spectroscopy (FTIR) spectra in Figure S5 of the Supporting Information, which demonstrates the weak vibration of C=O stretching, indicating the loss of oleic acid on the perovskite NCs. The short chain ligands could boost the self‐assembly process of NPLs to form cuboid NCs. The possible formation mechanism of the CsPbBr_3_ cuboid NCs through the assembly of multiple NPLs is depicted in Figure 2c. The CsPbBr_3_ NPLs are formed in the beginning stage of hot injection synthesis, and then self‐assemble side‐by‐side in order to decrease the free surface energy of the crystals. The ordered 2D NPLs of cuboid are isolated by OTA ligands.

**Figure 3:**
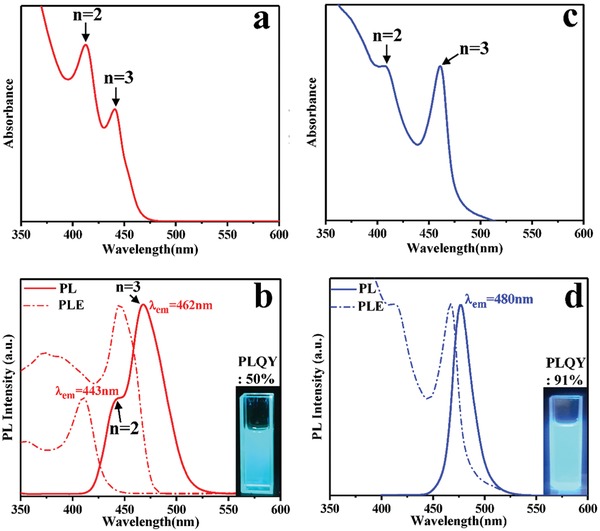
Optical absorption spectra of a) CsPbBr_3_ NPLs and c) CsPbBr_3_ cuboid NCs. PL (solid line) spectra of the b) CsPbBr_3_ NPLs with PLE (dashed‐dotted line) monitored at 443 and 462 nm, and d) CsPbBr_3_ cuboid NCs with PLE (dashed‐dotted line) monitored at 480 nm. The insets show photographs of the respective solutions under the excitation with a 365 nm UV lamp.

The UV–vis absorption spectra of the CsPbBr_3_ NPLs formed at the earlier stage of the reaction (10s) are given in **Figure** **3**a. The UV–vis spectrum potentially reveals the existence of two NPL species in the ensemble solution, with an absorption peak at 412 nm which can be assigned to the platelets consisting of 2 unit cells (and labeled *n* = 2 on the frame), according to previous literature,[qv: 10a] and another peak at 441 nm assigned to the platelets with 3 unit cells. Furthermore, the emission spectrum of such a bimodal mixture of NPLs is broad, and shows two PL peaks at 443 and 462 nm belonging to platelets with *n* = 2 and *n* = 3, respectively (Figure 3b).[qv: 10a] The blue‐shift spectra of CsPbBr_3_ NPLs compared with the general green‐emission CsPbBr_3_ NCs can be attributed to the quantum confinement effect due to the quantum confinement in one direction. The overall PL QY of these NPLs has been estimated as 50%. CsPbBr_3_ cuboid NCs exhibit a slightly blue‐shifted absorption shoulder at 407 nm, and a pronounced absorption peak which is strongly red‐shifted and appears at 463 nm (Figure 3c). Surprisingly, there is only one narrow, Gaussian‐shape emission peak (full width at half maximum—FWHM—equal to 21 nm) at 480 nm for the CsPbBr_3_ cuboid NCs (Figure 3d), with a PL QY as high as 91%, which is, to the best of our knowledge, the record value for any blue‐emitting perovskite NCs reported so far. Photoluminescence excitation (PLE) spectra of CsPbBr_3_ NPLs collected by monitoring PL at 443 and 462 nm exhibit two sharp, single, band‐edge peaks at 412 and 441 nm, respectively (Figure 3b), which are exactly at the same wavelengths as the respective absorption maxima in the UV–vis spectra (Figure 3a). This is a strong proof that the bimodal emission of NPLs originates from the mixture of platelets with different values of the unit cell (2 or 3). The low intensity at peaks ≈375 and ≈350 nm in PLE spectra of NPLs can be attributed to the high surface defect density of CsPbBr_3_ NPLs, revealing their limited ability to convert high‐energy photons. The PLE spectrum of cuboid NCs monitored at the emission peak of 480 nm shows two maxima at 410 and 469 nm (Figure 3d), which are somewhat red‐shifted from the respective positions of the absorption peaks (Figure 3c), indicating that the basic unit of photon absorption and emission in Cuboid NCs is still derived from single NPLs.

**Figure 4:**
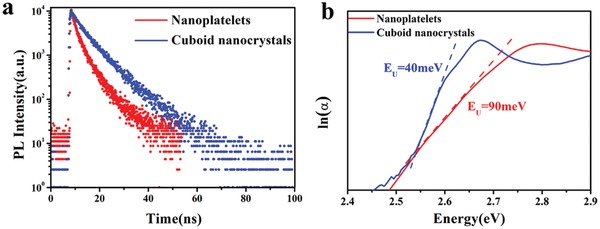
a) Time‐resolved PL decays of the CsPbBr_3_ NPLs (red) and the CsPbBr_3_ cuboid NCs (blue) and b) Urbach energy diagram for the CsPbBr_3_ NPLs and cuboid NCs.

Time‐resolved PL decay measurements were carried out to investigate in detail the recombination pathways in the CsPbBr_3_ NPLs and cuboid NCs. **Figure** **4**a presents the PL decay curves for the CsPbBr_3_ NPLs and cuboid NCs respectively, the excitation wavelength in both cases was 375 nm, and parameters of the respective three‐exponential fits are summarized in Table S2 of the Supporting Information. The emission from a monodisperse sample of perfect NCs without defects is expected to have a monoexponential decay, and eventually 100% PL QY. While the PL decay of the NPLs is clearly multi‐exponential, the decay of CsPbBr_3_ cuboid NCs is indeed very close to a single‐exponential (Figure 4a; Table S2, Supporting Information), indicating a much lower density of traps for the latter. The average PL lifetime of the NPLs is 5.3 ns, which is significantly shorter than that of CsPbBr_3_ cuboid NCs (8.6 ns), which again points toward the existence of a defect‐related exciton trapping process in NPLs. For the CsPbBr_3_ cuboid NCs, the self‐assembly process of NPLs results in the reduction of traps, thus efficiently eliminating a majority of nonradiative recombination pathways and resulting in improved PL QY from ≈50% to 91%. Compared to the radiative (9.4 × 10^7^ s^−1^) and apparent nonradiative (9.4 × 10^7^ s^−1^) decay rates of the NPLs, CsPbBr_3_ cuboid NCs show slightly faster radiative (10.5 × 10^7^ s^−1^) and much slower apparent nonradiative (1 × 10^7^ s^−1^) decay rates (Table S2, Supporting Information). For the apparent nonradiative rate we have neglected any dark fraction of emitters so the true nonradiative rates will be somewhat lower. We therefore have to concede that any change in the simple apparent nonradiative rate, may be due to a change in the true underlying nonradiative rate, or of the dark fraction of emitters, or both.

The changes in the recombination rates (the apparent nonradiative rate in particular) are most likely attributed to the fact that the CsPbBr_3_ cuboid NCs have lower trap state density. Some of the amplitude of the three components of the multiexponential fits as presented in Table S2 of the Supporting Information may originate from the short lifetime tail of a general broad distribution of emission lifetimes. In the case of CsPbBr_3_ NPLs, both the middle and long components are parts of the same broad lifetime distribution. In the case of CsPbBr_3_ cuboid NCs, the long lifetime may correspond to the delayed emission from the shallow traps.^13^ Since the PL QY in CsPbBr_3_ cuboid NCs is 91% and the amplitude of the trapping‐related fast component is 6%, the nonradiative decay yield from the shallow traps is small. This can be further visualized by plotting the absorption coefficient as a function of photon energy (Figure 4b); on this kind of diagram, the absorption edge is denoted as an Urbach tail: samples with few defects exhibit a negligible Urbach tail and a small Urbach energy *E*_U_. Urbach energies of CsPbBr_3_ NPLs and cuboid NCs are provided on Figure 4b: *E*_U_ of CsPbBr_3_ cuboid NCs is 40 meV, much smaller than that of the NPLs (90 meV).

**Figure 5:**
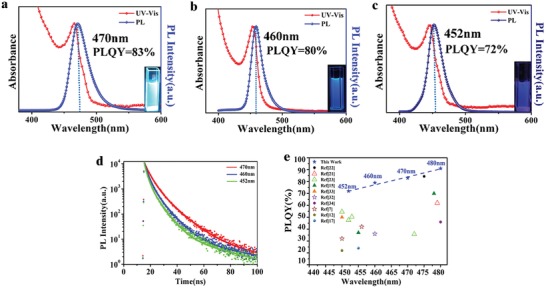
UV–vis and PL spectra of the CsPb(Br/Cl)_3_ cuboid NCs with the emission peaks centered at a) 470 nm, b) 460 nm, and c) 452 nm, which corresponds to the increasing content of Cl^−^. The insets show photographs of the respective solutions under the excitation with a 365 nm UV lamp. d) Time‐resolved PL decays (solid lines show multiexponential fits) of the CsPb(Br/Cl)_3_ cuboid NCs with the emission peak centered at 470, 460, and 452 nm. e) Summary of the emission peak positions and PL QYs of the blue CsPbBr_3_ and CsPb(Br/Cl)_3_ cuboid NCs compared with the respective data for blue‐emitting perovskite NCs reported in the previous literature.

As already discussed in the Introduction, the incorporation of Cl^−^ anions in the CsPbBr_3_ perovskite lattice may further expand the bandgap, and thus shift the PL peak to even shorter wavelengths, satisfying the demand for blue light emitters for displays (450–460 nm). Using the same synthetic strategy, we succeeded in making blue‐emitting CsPb(Br/Cl)_3_ cuboid NCs simply by introducing Cl^−^ ions into the reaction mixture, using different Br‐to‐Cl molar feeding ratios, namely 3:1, 2.5:1, and 2:1 (see the Supporting Information for details). Quantitative XPS analysis was carried out to confirm Br‐to‐Cl molar of CsPb(Br/Cl)_3_ cuboid NCs with the emission peak of 470, 460, and 452 nm, which demonstrated the Br/Cl ratio of 2.8/1, 2.5/1, and 1.9/1, respectively (Table S3, Supporting Information). The size and shape of the cuboid NCs made with an introduction of Cl^−^ was similar to that of the CsPbBr_3_ cuboid NCs (Figure S6, Supporting Information). The crystal sizes fitted by XRD and TEM as shown in Table S1 and Figure S3 (Supporting Information) are in good agreement (≈50 nm × 50 nm × 20 nm). The crystal structure of the CsPb(Br/Cl)_3_ cuboid NCs can be derived from their XRD patterns shown in Figure S7 of the Supporting Information, where the positions of the diffraction peaks correspond to orthorhombic CsPb(Br/Cl)_3_. As expected, XRD peaks shift to higher angles with an increasing amount of incorporated Cl^−^, due to the overall shrinking of the crystal unit cells. Similarly, a remarkable diffraction peak at 2θ = 22° in mixed‐anion cuboids XRD spectra was observed. We speculate that the incorporation of the Cl^−^ would not break the tendency of the orientation growth during the formation of the CsPb(Br/Cl)_3_ NPLs and following self‐assembly. Part of NPLs grows along the (110) crystal plane and thus this plane is exposed in the XRD spectra. **Figure** **5**a–c shows UV–vis and PL spectra of the mixed‐anion CsPb(Br/Cl)_3_ NCs, which possess narrow, Gaussian‐shaped emission peaks at 470, 460, and 452 nm, with FWHM of 26, 24, and 23 nm, respectively, and maintain high PL QY in the range of 83–72%. It is noted that the asymmetric band in their PL spectra can be attributed to the band tail state due to lattice mismatch after introducing Cl^−^.^14^ PL decay curves of these NCs are shown in Figure 5d; the average PL lifetimes obtained from the three‐exponential fittings (Table S4, Supporting Information) decrease from 8.7 to 7.8 ns upon increasing the content of Cl^−^. Radiative decay rates of the CsPb(Br/Cl)_3_ NCs with variable Cl content remain relatively constant ((9.2–9.4) × 10^7^ s^−1^), while the apparent nonradiative decay rates gradually increase from 1.9 × 10^7^ to 3.6 × 10^7^ s^−1^ with increasing the content of Cl^−^, which occurs hand‐in‐hand with decreasing PL QYs (Table S4, Supporting Information). These results overall indicate that the mixed‐anion CsPb(Br/Cl)_3_ NCs can still maintain low‐trap state density after the introduction of Cl^−^ anions, and provide blue emission with high PL QYs and a tunable peak wavelength over the broad spectral range relevant for display applications. Also, the CsPbX_3_ cuboid NCs exhibit excellent long‐term stability as shown in Figure S8 of the Supporting Information. CsPbBr_3_ and CsPb(Br/Cl)_3_ cuboid NCs solutions maintain a nearly constant PL QYs (>90%) after storing for 45 days under ambient conditions with the humidity of 60% at 25 °C (Figure S8a, Supporting Information). The solid‐state thin films based on the cuboid NCs show the excellent long‐term stability, which still retain the initial PL QYs of 80% after storing for 10 days (Figure S8b,c, Supporting Information).

There are several optical effects which can take place when NPLs assemble into cuboid NCs. First, *N* emitters placed close to each other may form a single emitter and exhibit the super‐radiance effect, i.e., to feature *N*‐fold enhancement in radiative decay rate.^15^ We can exclude manifestation of the super‐radiance in our experiments since we observed enhancement of luminescence efficacy without enhancement in the decay rate. Second, proximate displacement of emitters may result in fluorescence resonance energy transfer (FRET) provided there is an overlap between emission and absorption spectra of NPLs. In particular, FRET would lead to an emission red shift and to the narrowing of the emission spectrum of cuboid NCs. These effects have been widely reported for closely packed ensembles of II–VI quantum dots.^16^ Energy transfer may indeed occur in cuboid crystals owing to the overlap of the emission band for platelets with *n* = 2 and absorption spectrum of platelets with *n* = 3 (Figure 3c,d), as well as the short distance between the adjacent NPLs constituting the cuboid NCs. There would be no (or little) FRET in the solution ensemble of NPLs, due to the long distance between them. Third, the dense arrangement of the NPLs in a cuboid crystal may resemble to large extent semiconductor epitaxial multiple quantum well structures or even superlattices with certain disorder involved. In this case, the formation of minibands may result in the red shift of the absorption spectrum with electron and hole mobility over an ensemble whereas possible imperfections (disorder) may lead to localization of excitons in the local potential minima thus resulting in higher PL QY since most of the traps may not contribute to recombination processes.

The fourth effect is a possible contribution of reabsorption. By comparing Figure 3c,d one can see that for cuboids, the excitation spectrum shows a pronounced red shift with respect to the absorption spectrum. Reabsorption is known to result both in the emission red shift and in longer PL decay due to photon “recycling”^17^ and it has been extensively discussed recently in the context of bulk perovskites.^18^ One can see that the absorption and emission red shifts and longer PL decay in the cuboid NCs consisting of the densely arranged NPLs may be the consequence of different phenomena. However, our finding that the longer PL decay correlates with higher PL QYs toward the exciton localization on potential disorder sites as a preferential effect leading to the high PL QY of the cuboid crystals, helping localized exciton to bypass nonradiative channels present in other NPLs. However, we cannot exclude that the dense arrangement of NPLs modifies trap states on their surface thus avoiding fast nonradiative recombination to provide simultaneously longer PL decay close a monoexponential law, and a higher PL quantum yield. Self‐assembly of the NPLs into cuboid NCs possibly modifies their surface states responsible for traps formation. In the self‐assembly process, thinner and higher defect density NPLs may be dissolved, while the surface defects of the large NPLs can be healed. In addition, the individual NPLs of cuboids are completely isolated from each other by organic ligands, (as shown in Figure 1) so the cuboids exhibit blue emission. This is indirectly confirmed by the smaller Stokes shift of CsPbBr_3_ cuboid NCs (0.11 eV) compared with that of the NPLs (0.18 eV) as can be seen in Figure 3, suggesting that the cuboid NCs have fewer surface traps than NPLs.

## 3 Conclusion

In summary, we e present a synthetic strategy to produce regular‐sized (≈50 nm × 50 nm × 20 nm) CsPbX_3_ (X = Br or Br/Cl) cuboid NCs with an internal structure of multiple quantum wells, via a modified hot injection method by substituting the OAm ligand with OTA. The use of a relatively short OTA ligand with an eight‐carbon chain leads to the formation of 2D CsPbX_3_ NPLs at the initial reaction stage, which then undergoes spontaneous self‐assembly into larger CsPbX_3_ NCs with a cuboidal shape. These cuboids consist of multiple‐stacked NPLs isolated by OTA ligands, as directly confirmed by small‐angle X‐ray diffraction. Remarkably, CsPbX_3_ cuboid NCs demonstrate an exceptionally high PL QY of 91% at 480 nm, and a longer PL decay, which is tentatively ascribed to the effects previously observed in multiple quantum well structures, where exciton localization on potential disorder sites helps them to bypass nonradiative channels present in other NPLs. Using the same synthetic strategy while introducing some amount of Cl^−^ anions into the reaction mixture, mixed‐anion CsPb(Br/Cl)_3_ cuboid NCs with emission peaks centered between 452 and 470 nm, and considerably high PL QYs in the range of 72–83% have been produced. Emission peak positions and PL QY values of the PL QY of CsPbX_3_ (X = Br, or Br/Cl) cuboid NCs synthesized in this work are summarized and compared with previous data on blue‐emitting perovskite NCs in Figure 5e.[qv: 6a,10a,19] The PL QYs appear consistently higher than in the previous reports, which is one of the major advantages of the synthetic procedure introduced here. Blue‐emitting cuboid perovskite NCs based on multiple quantum wells demonstrated here offer significant potential for advanced lighting and displays.

## 4 Experimental Section

*Materials*: Cesium carbonate (Cs_2_CO_3_, 99.9%), lead(II) bromide (PbBr_2_ 98%), lead(II) chloride (PbCl_2_ 98%), OA (analytical reagent 90%), OAm (Aladdin 90%), octanoic acid (Aladdin 90%), OTA (Aladdin 90%), 1‐octadecene (ODE, technical grade 90%), toluene (anhydrous 99.8%), hexane (analytical reagent 97%), tert‐butanol (t‐BuOH, Aladdin 98%), and methyl acetate (MeOAc, anhydrous 99.5%) were used as received without further purification.

*Synthesis of Cs‐Oleate*: 0.1 g of Cs_2_CO_3_, 0.625 mL OA, and 10 mL ODE were loaded into a 100 mL 3‐necked flask and stirred under vacuum for 1 h at 120 °C. The flask was purged with N_2_ for 15 min, and the cycle of exchanging vacuum and N_2_ was repeated three times in order to remove any moisture and oxygen completely. The temperature of the solution was raised to 120 °C under N_2_ until it becomes clear, indicating that Cs_2_CO_3_ in ODE had fully reacted with OA. The clear Cs‐oleate solution was stored in N_2_ and preheated to 100 °C before using for the following synthesis of perovskite NCs, as otherwise it would precipitate at room‐temperature.

*Synthesis of CsPbX_3_ Cuboid NCs*: The CsPbBr_3_ cuboid NCs were synthesized following the hot‐injection method reported in the previous work,[qv: 1h] with some modifications. 10 mL ODE and a specific amount of PbX_2_ (0.138 g of PbBr_2_ for the samples with an emission at 480 nm, 0.104 g of PbBr_2_, and 0.026 g PbCl_2_ for the samples with an emission at 470 nm, 0.099 g of PbBr_2_, and 0.030 g PbCl_2_ for the samples with an emission at 460 nm, and 0.092 g of PbBr_2_ and 0.035 g PbCl_2_ for the samples with an emission at 452 nm) were loaded into a 100 mL 3‐necked flask and dried under vacuum at 120 °C for 1 h. The flask was purged with N_2_, and 1 mL OA and 1 mL OTA (preheated at 70 °C) were injected. The flask was placed under vacuum until the PbX_2_ had completely reacted with OA and OTA, and then kept under constant N_2_ flow, while the temperature was raised to 130–160 °C. The Cs‐oleate solution (1.6 mL, 0.0625 m), prepared as described above, was swiftly injected and after 40∼60 s reaction and the solution was then cooled by immersing the flask into an ice bath.

*Purification of CsPbX_3_ Cuboid NCs*: The CsPbX_3_ cuboid NCs were separated from the crude solution by adding t‐BuOH (volume ratio 1:1) and centrifugation at 9000 rpm for 5 min. The precipitate was collected and redispersed in hexane. This procedure was repeated three to five times, after which the precipitate was redispersed in toluene or hexane for further characterization.

*Characterization*: TEM and HRTEM images were collected on a JEOL JEM‐2010 microscope operated at 200 kV. X‐ray powder diffraction (XRD) was performed on an MXP21VAHF X‐ray diffractometer using Cu Kα radiation (λ = 1.5418 Å). UV–vis absorption spectra were collected on Beituo DUV‐18S2 and Shimadzu UV‐3600 plus spectrophotometers. XPS was carried out by a PHI5000VersaProbe III spectrometer. FTIR spectra were recorded on a Varian 3100 FTIR spectrometer. Steady‐state photoluminescence (PL) emission and PL excitation spectra were measured on a Gangdong F‐280 fluorescence spectrometer. PL QYs were estimated using an integrating sphere (Edinburgh, FLS920). Time‐resolved PL decays were recorded at room temperature on a Horiba Fluorolog spectrometer coupled with a 375 nm, 45 ps pulsed laser, and a time‐corrected single‐photon counting system. PL decays were fitted by a three‐exponential function, and the average PL lifetimes, radiative and apparent (i.e., estimated without a knowledge of the dark fraction of fluorophore) nonradiative rates were calculated based on standard procedures.^2^ Urbach energies *E*_U_ have been extracted by fitting the exponential part of the Urbach tail (see Figure 4b) according to ref. 7.

## Conflict of Interest

The authors declare no conflict of interest.

## Supporting information

Supplementary
