# Supplementary material for: Spontaneous Self‐Assembly of Cesium Lead Halide Perovskite Nanoplatelets into Cuboid Crystals with High Intensity Blue Emission
Source: Adv Sci (Weinh). 2019 May 8;6(13):1900462. doi: 10.1002/advs.201900462 (PMC6662087; doi:10.1002/advs.201900462)
Supplement: Supplementary file 1 — Supplementary [file ADVS-6-1900462-s001.pdf]

Copyright WILEY-VCH Verlag GmbH & Co. KGaA, 69469 Weinheim, Germany, 2019.

ADVANCED SCIENCE
Open Access

Supporting Information

for *Adv. Sci.,* DOI: 10.1002/advs.201900462

Spontaneous Self-Assembly of Cesium Lead Halide Perovskite Nanoplatelets into Cuboid Crystals with High Intensity Blue Emission

Chenghao Bi, Shixun Wang, Stephen V. Kershaw, Kaibo Zheng, Tönu Pullerits, Sergey Gaponenko, Jianjun Tian, and Andrey L. Rogach**

Copyright WILEY-VCH Verlag GmbH & Co. KGaA, 69469 Weinheim, Germany, 2019.

Supporting Information

Spontaneous Self-Assembly of Cesium Lead Halide Perovskite Nanoplatelets into Cuboid Crystals with High Intensity Blue Emission

Chenghao Bi, Shixun Wang, Stephen V. Kershaw, Kaibo Zheng, Tönu Pullerits, Sergey Gaponenko, Jianjun Tian, and Andrey L. Rogach**

C. Bi, S. Wang, Prof. J. Tian
Institute for Advanced Materials and Technology, University of Science and Technology Beijing, 100083, China.
E-mail: tianjianjun@mater.ustb.edu.cn

S. Wang, Dr. S. V. Kershaw, Prof. A. L. Rogach
Department of Materials Science and Engineering and Centre for Functional Photonics (CFP), City University of Hong Kong, Kowloon, Hong Kong SAR
E-mail: andrey.rogach@cityu.edu.hk (A.L.R.)

Dr. K. Zheng, Prof. T. Pullerits
Department of Chemical Physics and NanoLund, Lund University, P.O. Box 124, 22100 Lund, Sweden

Prof. S. Gaponenko
B.I. Stepanov Institute of Physics, National Academy of Sciences of Belarus, 68 Nezaležnasci Ave., 220072, Minsk, Belarus

ORCID
Stephen V. Kershaw: 0000-0003-0408-4902
Jianjun Tian: 0000-0002-4008-0469
Andrey L. Rogach: 0000-0002-8263-8141

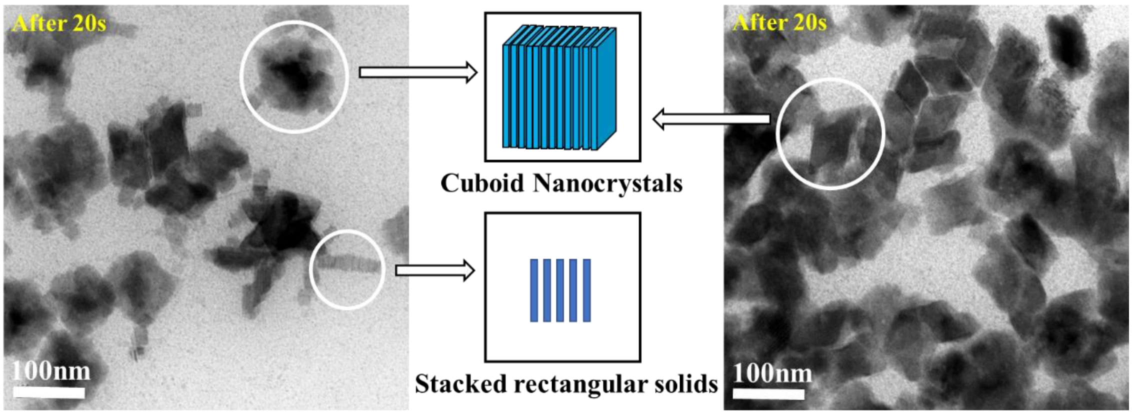

Figure S1. TEM images of intermediate products of the synthesis of $CsPbBr_3$ cuboid NCs.

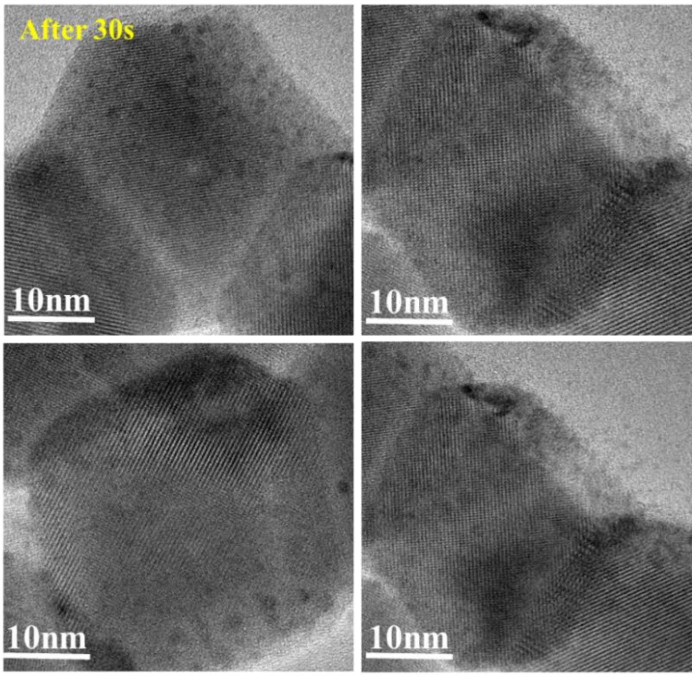

Figure S2. HRTEM images of representative $CsPbBr_3$ cuboid NCs recorded from different perspectives.

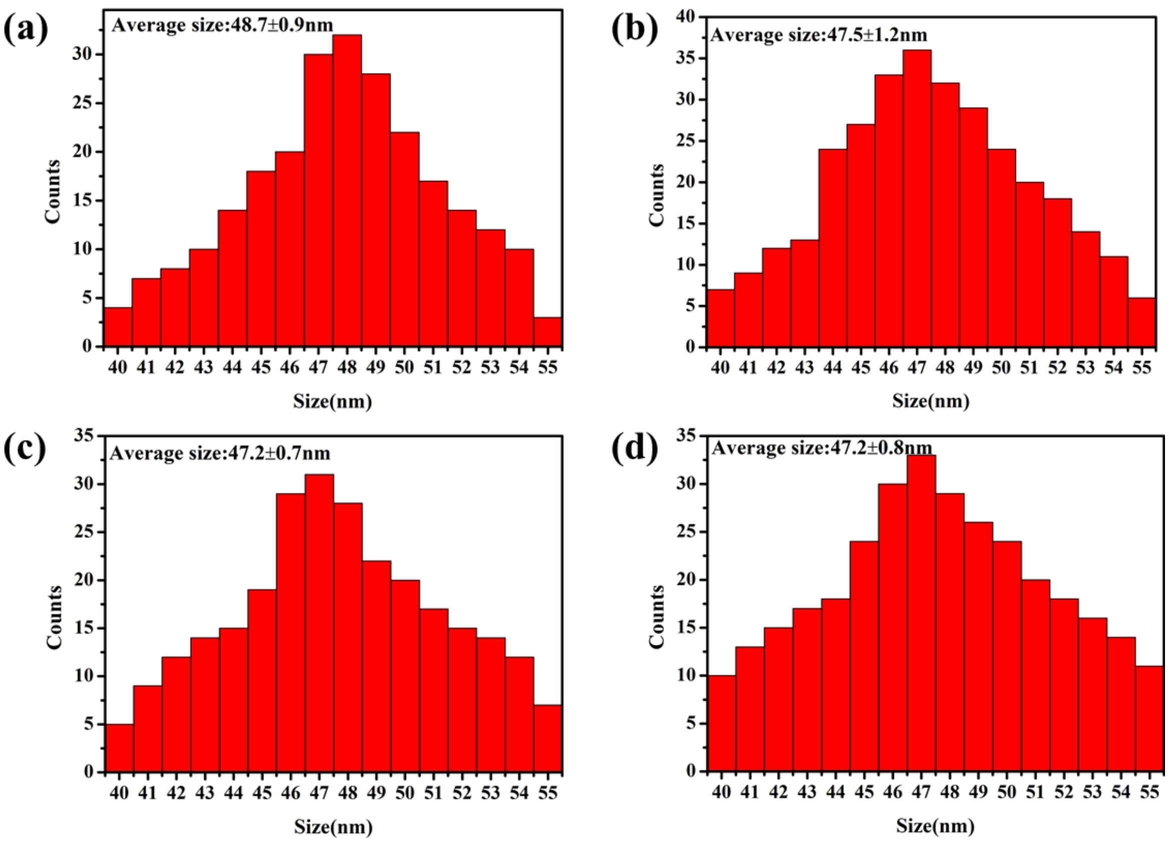

Figure S3. Size distribution histograms of **(a)** $CsPbBr_3$ **(b)** $CsPb(Br/Cl)_3$ (470 nm) **(c)** $CsPb(Br/Cl)_3$ (460 nm) (d) $CsPb(Br/Cl)_3$ (452 nm). The average sizes are calculated assuming that the data approximate to Gaussian distributions.

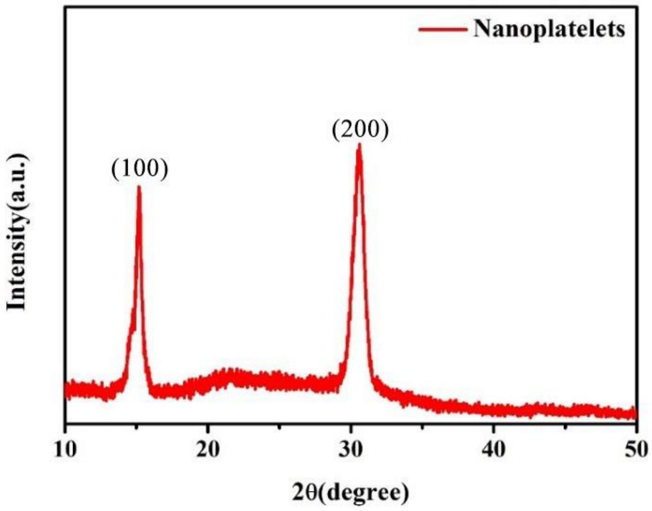

Figure S4. XRD pattern of $CsPbBr_3$ NPLs.

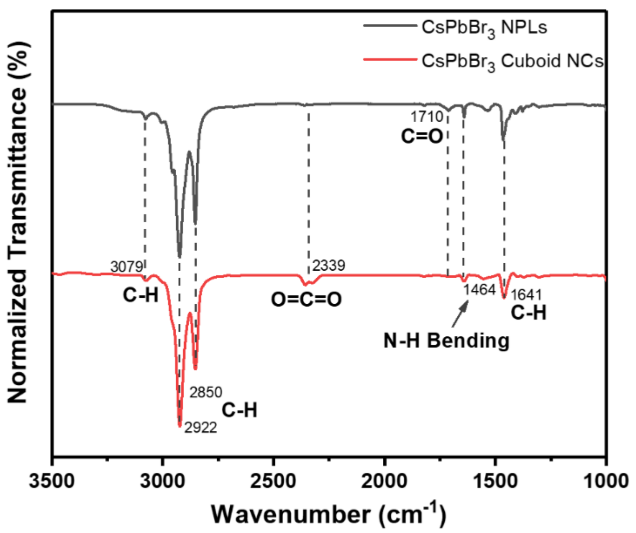

Figure S5. FTIR spectrum of the $CsPbBr_3$ NPLs and $CsPbBr_3$ cuboid NCs.

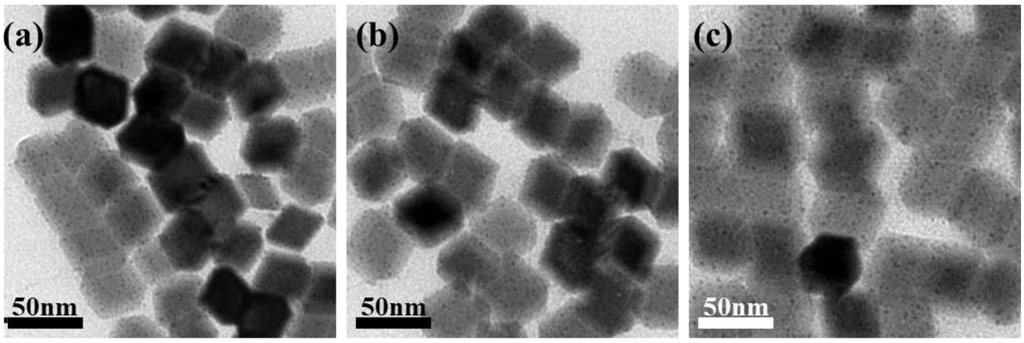

Figure S6. TEM images of the $CsPb(Br/Cl)_3$ cuboid NCs with emission peaks centered at **(a)** 470 nm, **(b)** 460 nm, and **(c)** 452 nm.

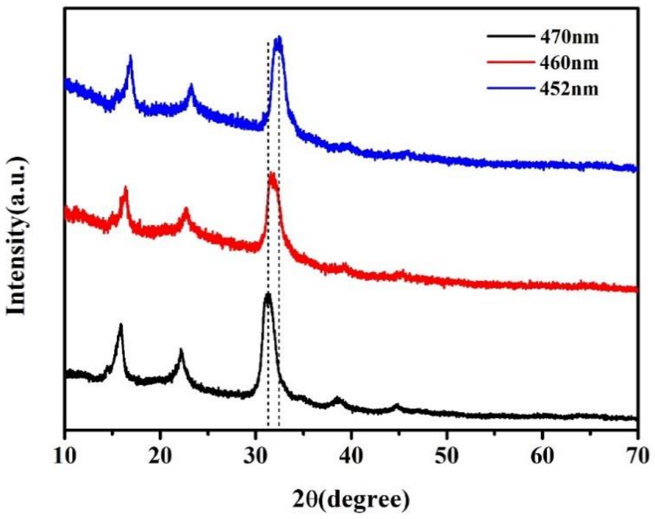

Figure S7. XRD patterns of the $CsPb(Br/Cl)_3$ cuboid NCs with emission peaks centered at 470 nm, 460 nm, and 452 nm.

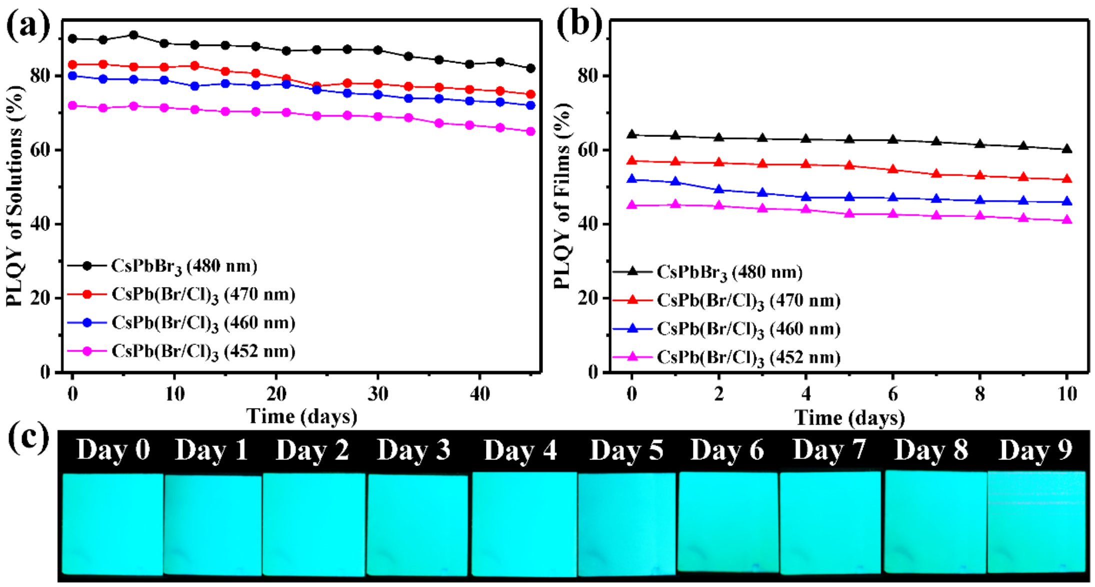

Figure S8. PL QYs of $CsPbX_3$ cuboid NCs **(a)** solutions **(b)** films with different storage time in ambient conditions (under an average humidity of 60%). **(c)** Photographs $CsPbBr_3$ cuboid NCs films (under 365 nm UV-light) after different stroage time.

Table S1. The crystal size of $CsPbX_3$ cuboid NCs estimated from XRD patterns

	$CsPbBr_3$	$CsPb(Br/Cl)_3$ (470 nm)	$CsPb(Br/Cl)_3$ (460 nm)	$CsPb(Br/Cl)_3$ (452 nm)
size (nm)	48.1±1.2	47.2±1.0	47.0±1.5	46.9±0.8

Table S2. Summary of three-exponential fitting parameters for time-resolved PL decays of $CsPbBr_3$ NPLs and cuboid NCs. The deviation of $K_r \times 10^7\ s^{-1}$ is calculated when the PL QY uncertainty is +/-0.5% and that of the lifetimes is +/-0.1ns.

$CsPbBr_3$	A_1 [%]	τ_1 [ns]	A_2 [%]	τ_2 [ns]	A_3 [%]	τ_3 [ns]	τ_{avg} [ns]	PLQY [%]	$K_r \times 10^7$ [s^{-1}]	*apparent* $K_{nr} \times 10^7$ [s^{-1}]
NPLs	10	0.9	50	3.2	40	8.9	5.3	50	9.4±0.3	9.4±0.3
Cuboid NCs	6	1.3	87	8.3	7	19	8.6	91	10.5±0.2	1.0±0.2

Table S3. Quantitative XPS analysis results of $CsPbX_3$ cuboid NCs.

Cuboid NCs	Cs [%]	Pb [%]	Br [%]	Cl [%]
$CsPbBr_3$	19.8	20.2	60.0	0
$CsPb(Br/Cl)_3$ (470 nm)	19.2	20.7	44.3	15.8
$CsPb(Br/Cl)_3$ (460 nm)	19.5	19.9	43.3	17.3
$CsPb(Br/Cl)_3$ (452 nm)	19.4	20.5	39.4	20.7

Table S4. Summary of three-exponential fitting parameters for time-resolved PL decays of the $CsPb(Br/Cl)_3$ cuboid NCs with emission peaks centered at 470 nm, 460 nm, and 452 nm. The deviation of $K_r \times 10^7\ s^{-1}$ is calculated when the PL QY uncertainty is +/-0.5% and that of the lifetimes is +/-0.1ns.

PL peak	A_1 [%]	τ_1 [ns]	A_2 [%]	τ_2 [ns]	A_3 [%]	τ_3 [ns]	τ_{avg} [ns]	PLQY [%]	$K_r \times 10^7$ [s^{-1}]	*apparent* $K_{nr} \times 10^7$ [s^{-1}]
470 nm	11.7	0.7	61.3	5.9	27.0	20	9.1	83	9.1±0.2	1.8±0.2
460 nm	14.3	1.9	59.4	5.8	26.3	19.8	8.9	80	8.9±0.2	2.2±0.2
452 nm	20.3	1.7	58.2	6.1	21.5	19.5	8.1	72	8.8±0.2	3.5±0.2
